# First Neuromuscular Contact Correlates with Onset of Primary Myogenesis in Rat and Mouse Limb Muscles

**DOI:** 10.1371/journal.pone.0133811

**Published:** 2015-07-24

**Authors:** Bradley Hurren, Jennifer J. P. Collins, Marilyn J. Duxson, Marianne Deries

**Affiliations:** 1 Department of Anatomy, University of Otago, Dunedin, New Zealand; 2 Regenerative Medicine Program, Ottawa Hospital Research Institute, Ottawa, Ontario, Canada; 3 Centro de Evolução, Ecologia e Alterações Ambientais, Faculdade de Ciências, Universidade de Lisboa, Lisbon, Portugal; University of Minnesota Medical School, UNITED STATES

## Abstract

Skeletal muscle development has been the focus of intensive study for many decades. Recent advances in genetic manipulation of the mouse have increased our understanding of the cell signalling involved in the development of muscle progenitors which give rise to adult skeletal muscles and their stem cell populations. However, the influence of a vital tissue type – the peripheral nerve—has largely been ignored since its earliest descriptions. Here we carefully describe the timing in which myogenic progenitors expressing Pax3 and Pax7 (the earliest markers of myogenic cells) enter the limb buds of rat and mouse embryos, as well as the spatiotemporal relationship between these progenitors and the ingrowing peripheral nerve. We show that progenitors expressing Pax3 enter the limb bud one full day ahead of the first neurites and that Pax7-expressing progenitors (associated with secondary myogenesis in the limb) are first seen in the limb bud at the time of nerve entry and in close proximity to the nerve. The initial entry of the nerve also coincides with the first expression of myosin heavy chain showing that the first contact between nerves and myogenic cells correlates with the onset of myogenic differentiation. Furthermore, as the nerve grows into the limb, Pax3 expression is progressively replaced by Pax7 expression in myogenic progenitors. These findings indicate that the ingrowing nerve enters the limb presumptive muscle masses earlier than what was generally described and raises the possibility that nerve may influence the differentiation of muscle progenitors in rodent limbs.

## Introduction

This paper establishes, for the first time, that the very early muscle masses of mammalian limb buds, composed largely of undifferentiated muscle precursor/progenitor cells (MPCs), develop in the presence of innervation. Why is this important, and did we not know this already?

Skeletal muscle development has been a key model system in the field of developmental genetics, so it is important that the model includes consideration of all relevant factors. Not only internal genetic networks need to be elucidated, but also how those networks are affected by influences originating from surrounding tissues and physiological partners *in vivo*.

Developing skeletal muscle was the first system in which a single gene was shown capable of determining cell type [1–3]. Since then, a great deal has been learned about the genetic networks regulating myogenic development (see review [4]). All muscle cells—except those of the head—originate from the embryonic somites, and, in early development, gradients of morphogens from tissues surrounding the somites drive somitic cells towards or away from a myogenic fate [5–7]. Thus there is a complex interaction between the internal genetic network of the somitic cells and their immediate environment that ultimately determines whether they enter the myogenic fate or not.

Once myogenic cell fate is determined, the myogenic progenitor/precursor cells (MPCs) continue on to form distinct muscle masses. Within these masses, terminal differentiation is initiated by formation of mononucleated primary myocytes (at E13.5 in the mouse hindlimb) which subsequently nucleate formation of multinucleated myotubes [8]. During terminal differentiation, myogenic development is again intimately tied to that of other tissues, and particularly to that of the connective tissues and of muscle’s prime physiological partner, the nervous system. Developmental interactions between early myogenic cells and connective tissue have started to be elucidated and connective tissues shown to have critical roles in muscle patterning and in fibre type development [9–11]. However, the potential ability of the nervous system to influence the genetic cascades of early myogenic differentiation has, surprisingly, received little attention (but see [12], referred to below). This is especially surprising given what we know about the profound effects of neural input in later muscle development, including effects on the number of muscle fibres formed and in determining physiological fibre types [13–15]

Why does this gap exist? A major reason is that many studies of neuromuscular interactions in the developing chick limb, starting from the late 1970s, suggested that innervation of the muscle masses occurs at a relatively late stage of myogenic differentiation, when muscle fibres are already well formed and able to respond to electrical stimulation (eg: [16–18]). In fact, in the chick, entry of nerves into the muscles seems to be almost deliberately delayed. After initial outgrowth of motor and sensory axons to the region of the limb plexus, a ‘waiting period’ of 16–24 hours occurs, during which the muscle masses mature to form multinucleated muscle fibres while neural outgrowth remains frozen [19]. Transplantation experiments between embryos of different stages have shown that this waiting period is a function of the maturity of the limb structures–when more mature limb buds are grafted onto young hosts, the nerves can grow into the limbs immediately [20]. Thus, in the chick limb bud, it appears that early differentiation of muscles normally occurs in the absence of neural influence.

In the early 20^th^ century, many careful light microscopic studies in mammals also concluded that nerves did not invade the limb bud until quite late stages of development (eg: [21, 22] examining limb buds of human embryos), and the limb bud became regarded as a ‘self-organising structure’. However, Cameron & McCredie later re-examined this conclusion in rabbit embryos using more advanced methods of tissue fixation, serial 1 μm resin sections and electron microscopy [23]. Their paper showed clear images of well-developed nerve bundles entering the base of the limb bud at a time when the pre-muscle masses had not yet formed, then branching and infiltrating the masses as soon as they appeared. These observations were not much recognised by the research community, however, perhaps because the undifferentiated masses could not be positively identified as myogenic with electron microscopy. Around the same time, many electrophysiological and electron microscopic studies reported that initiation of formation of neuromuscular junctions within various developing mammalian muscles generally occurred much later, once the multinucleated primary myotubes of the muscle anlage were established (eg: rat diaphragm: [24]; mouse EDL: [25]; mouse soleus: [26]). Thus, despite the existence of contradictory evidence, the belief became widespread that penetration of axons into developing muscle occurs at a relatively late stage (eg: see reviews [27, 28]).

More recently, characterisation of the proteins Pax3 and Pax7, which identify myogenic precursor cells (see review [29]), has made it possible to visualise the developing muscle masses at much earlier developmental stages. Concurrently, many transgenic studies have incidentally shown clear images of nerves growing into the vicinity of developing muscles at very early times (eg: [30, 31]), but rarely has nerve ingrowth been systematically correlated with stage of muscle development. The myotome and its derivatives, the epaxial muscles, are an exception. In 2008, Deries et al [32] established that while the embryonic myotomal muscle of the mammal is never innervated, nerve growth cones enter the derivative epaxial muscle masses as soon as they begin to form. In a significant recent paper, Van Ho et al [12] further reported that contact between neural crest cells (the cells of origin of the peripheral nervous system) and somites regulates the balance between proliferation and differentiation of the MPCs destined to form the epaxial muscles in mice, although they did not see the same effect for the limb muscles. Innervation of muscles derived from the myotome is relatively delayed compared to that of the limb and other muscles of migratory origin [32] making it likely that interactions between myogenic cells and cells of the neural lineage may be different in the two types of muscle. Overall, it seems timely to establish a clear sequence of events correlating neural outgrowth with stage of muscle development within the developing mammalian limb.

Here, we make a systematic examination of when the outgrowing nerve and the developing MPCs first meet in the mammalian limb bud, using dual immunohistochemistry for nerve, and for all stages of myogenic cells, in various preparations of developing rodent limb buds. The results of this simple study suggest that mammalian limb myogenesis *in vivo* occurs in the presence of the nervous system from a time shortly after the formation of the dorsal and ventral masses, when they are formed almost entirely of MPCs, and well before muscle cleavage or the formation of multinucleated myotubes have commenced. In addition, while Pax3-positive MPCs are present in the muscle masses well before nerve ingrowth, appearance of Pax7-positive MPCs is spatially and temporally correlated with ingrowth of the nerves to the limb.

## Material and Methods

This study and the protocols used in the study were approved by the Committee for the Ethical Use of Animals in Research, University of Otago, Dunedin, New Zealand, permit numbers 15/09 and 25/09.

### Collection of rat and mouse embryos

Dated pregnancies were obtained from Wistar rats and C57BL/6 mice by the observation of copulation plugs, with the morning of the plug considered as embryonic day 0.5 (E0.5). Embryos were collected at half-day (12 hour) intervals from E12.5 to E15 for rat embryos and E11 to E11.5 for mouse embryos. The dam was anaesthetised with a mixture of ketamine (Monarch Pharmaceuticals; 100 mg/kg) and xylazine (Wyeth-Ayerst Veterinary Laboratories; 20mg/kg) delivered intraperitoneally, and deep anaesthesia confirmed by lack of response to a painful toe pinch and lack of corneal reflex. Embryos were then removed from the uterine horns, euthanased, and limb buds excised and processed for cryosectioning and immunohistochemistry, as below.

### Immunohistochemistry on sections

Limb buds were embedded according to the protocol of Bajanca et al [33]. Briefly, tissues were fixed in 0.2% paraformaldehyde (PFA) overnight, then incubated in a series of phosphate buffer and sucrose solutions of increasing concentration. They were then carefully oriented and frozen over liquid nitrogen in a gelatine/ sucrose/ phosphate buffer solution before storage in sealed containers at -80°C. Transverse cryosections of the embryo, at limb level (at either 10 μm or 20 μm) were collected on gelatine coated slides, dried and processed for immunohistochemistry according to the protocol of Deries et al [32]. Sections were lightly fixed in 0.2% PFA and blocked with 10% goat serum in 1% BSA/PBS before primary antibody incubation at 4°C (see Table 1 for details of antibodies). After several PBS washes at room temperature the sections were blocked again with 10% goat serum in 1% BSA/PBS and incubated with secondary antibodies either 1 hour at room temperature or overnight at 4°C. After more PBS washes the sections were fixed in 4% PFA and mounted in DABCO/glycergel or Vectashield.

**Table 1:** Antibodies.

**Primary antibodies**					
**Antigen**	**Antibody Type**	**Source**		**Cat #**	**Dilution**
**Embryonic MHC**	Mouse mAb: F59	DSHB (Iowa, US)		F59	1:25
**Pax3**	Mouse mAb: Pax3	DSHB (Iowa, US)		PAX3	1:100
**Pax7**	Mouse mAb: Pax7	DSHB (Iowa, US)		PAX7	1:20
**Pax7**	Rabbit pAb: Pax7	Aviva Sys. Biol. (San Diego, USA)		ARP32742_P050	1:200
**Neurofilament**	Rabbit pAb: NF145	Abcam (MA, US)		AB1987	1:1000
**Synaptophysin**	Rabbit pAb: Syn	Abcam (MA, US)		A0010	1:1000
**Secondary Antibodies**					
**Antigen / toxin**	**Conjugate**	**Species**	**Source**	**Cat #**	**Dilution**
**Mouse IgG**	Alexa Fluor 488 (F(ab’)_2_	Goat	Molec. Probes (NY, US)	A-11017	1:2000
**Mouse IgG**	Alexa Fluor 568 (F(ab’)_2_	Goat	Molec. Probes (NY, US)	A-11019	1:2000
**Rabbit IgG**	Alexa Fluor 488 (F(ab’)_2_	Goat	Molec. Probes (NY, US)	A-11070	1:2000
**Rabbit IgG**	Alexa Fluor 568 (F(ab’)_2_	Goat	Molec. Probes (NY, US)	A21069	1:2000
**α-bungarotoxin**	Alexa fluor 647		Molec. Probes (NY, US)	B35450	1:200

DSHB = Developmental Studies Hybridoma Bank

### Antibodies

Immunohistochemistry was used to visualize nerves, muscle precursor cells and differentiated muscle cells in transverse sections of rat and mouse embryonic forelimb and hindlimb buds. Nerves were detected with antibodies against neurofilament and synaptophysin, muscle precursor cells with antibodies against Pax3 and Pax7, and differentiated muscle cells with an antibody to myosin heavy chain (MHC). When mouse monoclonal antibodies against Pax3 and Pax7 are combined with the antibody against MHC and detected by the same secondary antibody, it is possible to distinguish the different labels because MHC is expressed in the cytoplasm and appears brighter than either Pax3 or Pax7 which are both transcription factors present in the nucleus.

### Imaging

Immunoreacted tissues were visualised and photographed either by fluorescence (Olympus BX50 fluorescent microscope) or confocal microscopy (Carl Zeiss laser scanning confocal microscope LSM 510). The digital image stacks obtained by confocal microscopy were analysed using ImageJ software (Rasband, W.S., ImageJ, U.S. National Institutes of Health, Bethesda, Maryland, USA) and Adobe Photoshop (Adobe Systems Incorporated, San Jose, CA, USA). Figures were produced with Adobe Illustrator (Adobe Systems Incorporated).

### Quantitation of Muscle Precursor Cell Numbers

Muscle precursor cell numbers were quantified in rat embryo hindlimbs at ages E12.5-E14.5. Cell counts of Pax3-positive and Pax7-positive cells were made throughout the entire hindlimb as follows. The limb bud was serially sectioned (12μm) in the transverse plane and every 5^th^ section analysed. A mylar counting grid was overlaid on images of these sections and all Pax3^+ve^ or Pax7^+ve^ cells within every mesh square were counted. Usually 4 to 5 sections were analysed for each limb bud, and the counts then pooled to give a number for that animal, with limb buds from 5 animals analysed at each age. Further to this, each image was analysed 3 separate times and an average of the counts taken to ensure that counting bias was eliminated. Prism (Graphpad Software Inc., La Jolla, CA, USA) was used for all data analyses and production of graphs.

Once these counts had been made, the image showing nerve staining was merged with the Pax3- or Pax7-immunoreacted image to show the spatial relationship between muscle precursor cells and innervation.

## Results

### Time course of nerve outgrowth in the limb muscle masses

**Fig 1:**
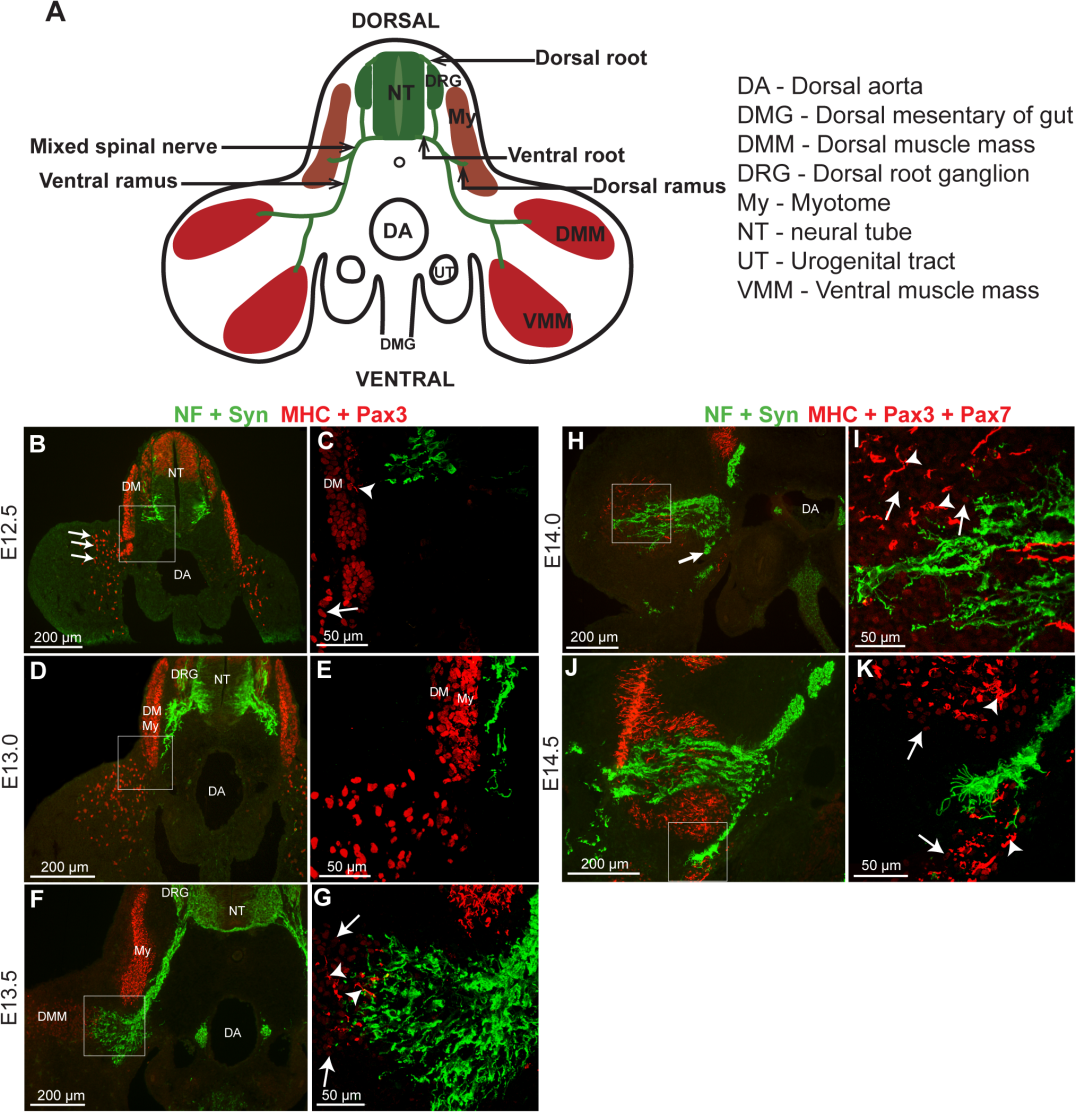
Time course of outgrowth of nerve towards the hindlimb muscle masses. **A** Schematic overview of the structures in a transverse section of the rat embryo at hindlimb level. **B**-**K** Fluorescent immunohistochemistry of 10 μm transverse sections of rat hindlimb, from E12.5 to E14.5. Sections immunoreacted with a mix of neurofilament (NF) and synaptophysin (Syn) antibodies to show the outgrowing nerve (green), and with a mix of myosin heavy chain (MHC) and Pax3 antibodies to show the muscle masses (red), at stages E12.5—E13.5 (**B**–**G**); or a mix of MHC, Pax3 and Pax7 antibodies at stages E14.0 and E14.5, (**H**–**K**). MHC is expressed in the cytoplasm (arrowheads in **C**, **G**, **I**, **K**), and its staining appears brighter than that of Pax3 and Pax7 which are present in the nuclei of MPCs (arrows in **C**, **G**, **I**, **K**). Images **B**, **D**, **F**, **H**, and **J** are from the fluorescent microscope, whereas **C**, **E**, **G**, **I**, **K** are confocal images. Pax3-positive MPCs invade the limb mesenchyme before the ventral nerve grows out (A-E). The ventral nerve reaches the differentiating muscle mass in the limb when it contains only a few differentiated MHC positive cells (F,G) and then quickly invades the early muscle mass (H-K). **B**, **C**: E12.5 **D**, **E**: E13.0 **F**, **G**: E13.5 **H**, **I**: E14.0. The arrow in **H** indicates an axon bundle branching towards the ventral muscle mass. **J**, **K**: E14.5. DM–dermomyotome, My—myotome, NT–neural tube, DRG–dorsal root ganglia.

The time course of outgrowth of the ventral ramus of the spinal nerves towards the muscle masses of the limb was visualised using a barrage of antibodies against the nerve (in green) and against both muscle precursor cells and myocytes/fibres (in red), so as to mark the entirety of each tissue (see methods for detail). Critically, the antibodies used to define the extent of the nerve included not only an anti-neurofilament antibody, which stains the core of the axon and pre-terminal axon, but also an antibody to synaptophysin, which detects an element in the axonal cytoplasm extending to the distal extremity of the growth cone (eg: [34]). Initial observations were of 10 μm serial transverse sections of rat hindlimbs between embryonic day (E) 12.5 and E14.5 at half-day intervals (Fig 1). An overview of the structures in a transverse section of rat embryo at hindlimb level is shown in Fig 1A.

At E12.5 (Fig 1B and 1C), the ventral root (motor) axons have just exited from the ventral aspect of the spinal cord; the neurons of the dorsal root ganglia (sensory) have not yet formed. The motor axons extend towards, but do not contact, the very early myotome, which is formed of only a few differentiated cells (arrowhead, Fig 1C). At this stage, the migrating MPCs are delaminating from the ventral lip of the dermomyotome (Fig 1C, arrow) and the first ones have reached the proximal part of the limb bud (Fig 1B, arrow), as previously described in Lee et al, 2013 [8].

By E13.0, the dorsal root ganglion has formed (Fig 1D, DRG) and sensory axons extend from it to join the motor nerves, forming the mixed spinal nerve. The ventral branch of this nerve (the ventral ramus) extends into the sclerotome and down towards the ventromedial boundary of the myotome, but as previously reported, the axons specifically avoid contact with cells of the growing myotome (Fig 1D and 1E) [32, 35]. Delamination of MPCs from the dermomyotome continues and Pax3-positive cells scatter throughout the proximal part of the hindlimb bud, but there is no spatial overlap between axons and limb muscle masses, and no extended MHC positive cells are yet apparent (Fig 1E).

Twelve hours later (E13.5, Fig 1F and 1G), the dorsal and ventral muscle masses of the hindlimb bud have formed and a profuse array of growth cones from the ventral spinal ramus extends just into the proximal edge of the dorsal muscle mass. (DMM, Fig 1F, and at higher magnification in 1G). A few differentiated myocytes/myofibres, defined by their extended linear shape and bright cytoplasmic staining for MHC, are now seen amongst the dimly stained Pax3-positive MPCs of the dorsal muscle mass, and occur predominantly in close proximity to the ingrowing nerve (arrowheads in Fig 1G). Note that, in marked contrast to what is seen in the chick [20, 36] we observe no sign of a hiatus in axonal growth at the ventromedial border of the dermomyotome.

Growth cones of the ventral ramus continue to extend and ramify profusely within the dorsal limb muscle, branching towards the ventral muscle mass by E14.0 (Fig 1H, arrow), and entering it at E14.5 (Fig 1J and 1K). By E14.5, the young muscle masses are occupied by an almost equal volume of ramifying nerve axons and growth cones. These growth cones are notably large, as seen in the confocal image of Fig 1K. The number of MHC expressing myocytes/myofibres has also steadily increased (arrowheads in Fig 1G, 1I and 1K) and at all times examined, they occur more frequently in regions close to the nerve.

**Fig 2:**
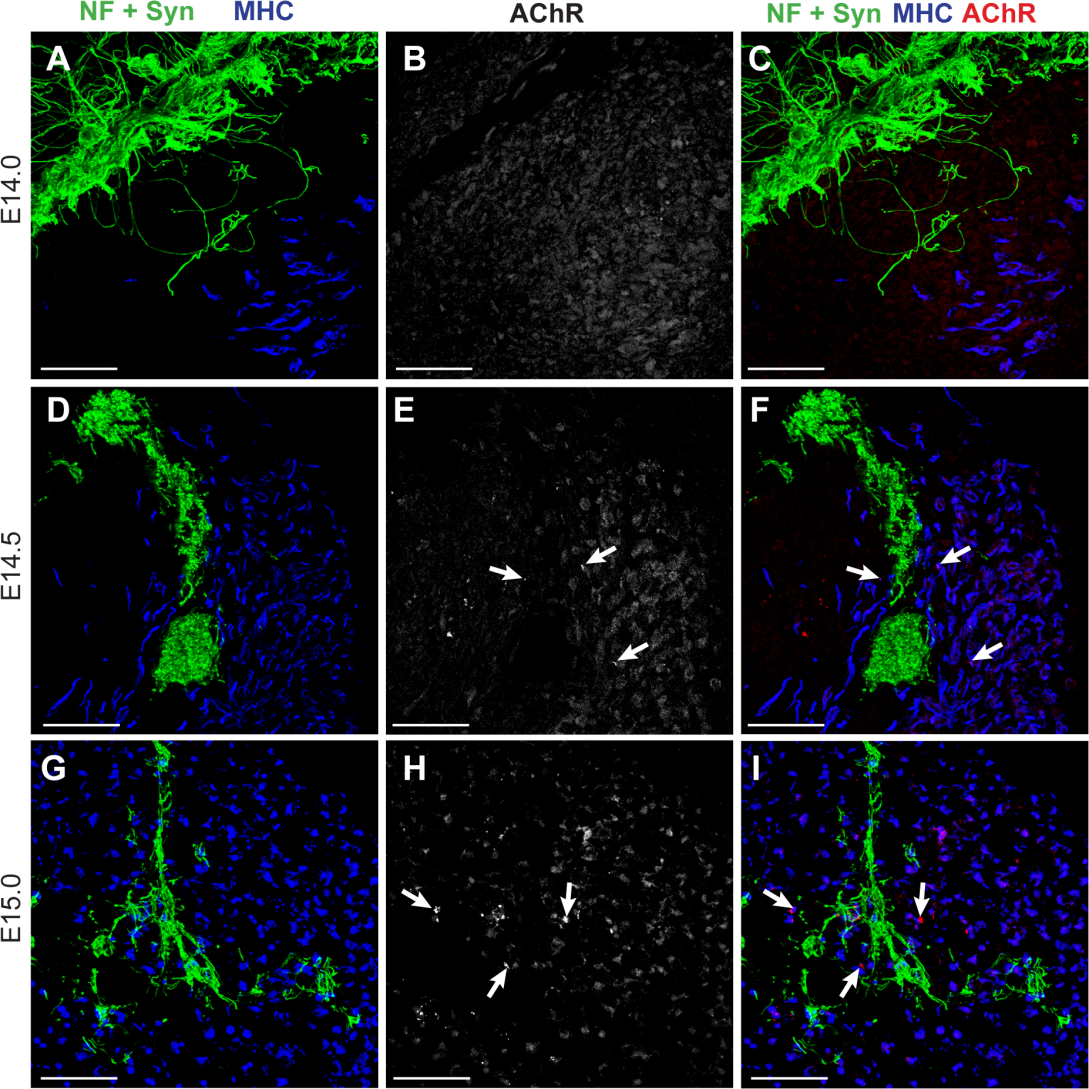
Appearance of AChR clusters. Z projections of confocal images from 20 μm transverse sections of rat hindlimbs between stages E14.0 and E15.0 stained by fluorescent immunohistochemistry with antibodies against neurofilament (NF) and synaptophysin (syn) marking nerve outgrowth (green) and against MHC (differentiated muscle cells, blue), and with α-bungarotoxin conjugated to Alexa fluor 647 (red) to show the acetylcholine receptors (AChR). AChR are first diffusely spread on muscle cells (E14.0, **A**-**C**). The first AChR clusters appear at E14.5 (arrows in **E** and **F**) and their number increases (arrows in **H** and **I**) as neurites invade the growing muscle mass. Scale bars = 50μm

To further characterise the relationship between nerve and myogenic cells in rat hindlimb muscles, we next examined the timing of appearance of acetylcholine receptors (AChR) and their clustering. The presence of AChR, either diffusely or in clusters, would indicate that myogenic cells are capable of responding to neurologically released AChR [37]. AChR were detected using α-bungarotoxin conjugated to the fluorophore Alexa 647 (Fig 2).

Appearance of AChR on the myogenic cells lagged behind entry of the nerve into the muscle masses. We first saw AChR distributed diffusely on the myogenic cells of the dorsal muscle mass at E14.0, about half a day after arrival of the nerve (Fig 2A–2C). The first few defined AChR clusters were seen at E14.5 (Fig 2E and 2F arrows) and by E15.0 they were numerous throughout the developing limb muscles (Fig 2H and 2I, arrows).

Thus, invasion of the rat hindlimb muscle masses by the nerve first occurs at E13.5, almost exactly co-incident in time and space with the very beginning of myogenic differentiation. From E14.0, nerve and muscle cells may in theory communicate with each other via acetylcholine released from growth cones (eg: [38]) as AChR are already present at this stage, although distinct AChR clusters do not appear until E14.5.

We have further confirmed that the same sequence of events occurs in the forelimb of the rat, but half a day earlier than in the hindlimb (S1A and S1B Fig and data not shown), and in both fore- and hindlimbs of the mouse (S2A, S2B, S2I and S2H Fig and data not shown).

### Spatial and temporal relationships between invading nerve and limb MPCs

We have previously shown that Pax3- and Pax7-positive MPCs have different temporal and spatial distributions in the early muscle masses of the rat hindlimb [8]. Here we examine whether they also have a different relationship to the ingrowing muscle nerves.

**Fig 3:**
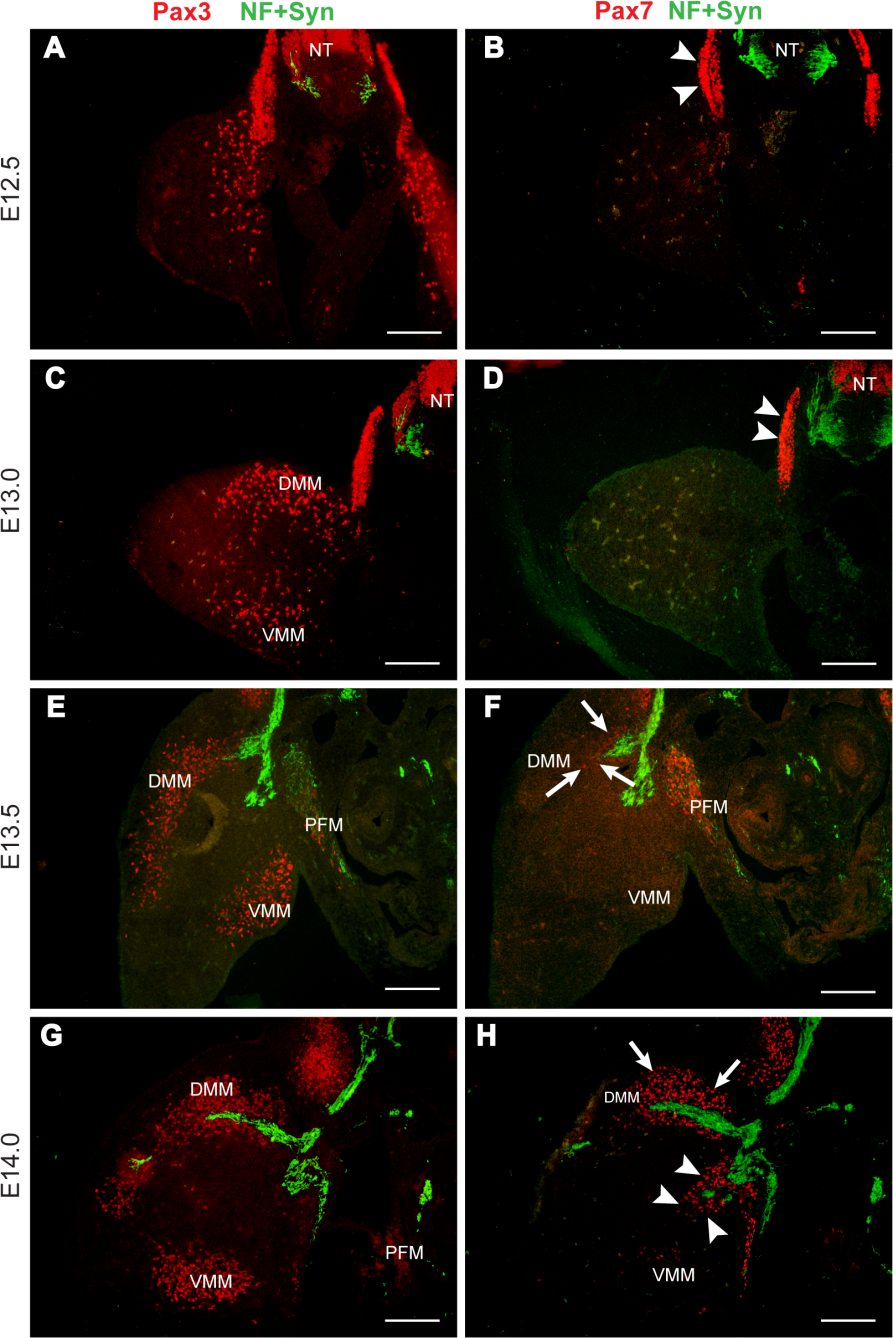
Temporal relationship between Pax3- and Pax7-positive MPCs and the innervating nerve. Fluorescent microscopic images of serial pairs of 10 μm transverse sections of rat embryos at hindlimb level, between stages E12.5 and E14.0. Sections were immunoreacted with antibodies against neurofilament (NF) and synaptophysin (syn) to show the nerve (green), and either Pax3 (**A**, **C**, **E**, **G**) or Pax7 (**B**, **D**, **F**, **H**) in red. Pax7 immunoreactivity is absent from the limb at early stages, although high in the nearby dermomyotome (**B**, **D**—arrowheads), whereas Pax3-positive MPCs have already entered the limb by E12.5 (**A**, **C**). At E13.5, a population of Pax7-positive MPCs appear in the hindlimb, closely associated with the site of ingrowth of the limb nerve (**F**—arrows). Pax7 immunoreactivity then expands within the dorsal muscle mass (**H**—arrows) and spreads along the ventral branch of the limb nerve (arrowheads) towards the ventral muscle mass. DMM = dorsal muscle mass, VMM = ventral muscle mass, PFM = pelvic floor muscle, NT = neural tube. Scale bars = 200 μm.

Pax3-positive MPCs first enter the hindlimb at E12.5 (Fig 3A), are initially dispersed throughout the dorsal to ventral boundaries of the proximal limb bud, then by E13.0 begin to segregate into dorsal and ventral muscle masses (Fig 3C–DMM and VMM). These steps clearly precede ingrowth of the muscle nerves (Fig 3A and 3C), and the early MPCs are not immunoreactive for Pax7 (Fig 3B and 3D) as reported in Lee et al 2013 [8]. As a control for the immunohistochemistry, strong expression of Pax7 is seen in cells of the dermomyotome at these same times (Fig 3B and 3D—arrowheads).

Pax7-positive MPCs are first seen within the limb one full day later, at E13.5, in close proximity to the invading limb nerve (Fig 3F—arrows). They initially lie only within the most proximal part of the DMM, while the Pax3-positive MPCs have established clear dorsal and ventral muscle masses (Fig 3E). As reported in the previous section, the nerve has now invaded the DMM, but the VMM remains uninnervated, as well as lacking in Pax7 reactivity. Subsequently (E14.0), Pax7-positive MPCs spread further into the limb bud, appearing to flow along a path very close to that of the nerve as they both extend towards the VMM (Fig 3H). Comparing Fig 3G and 3H, the two populations overlap spatially to a moderate extent in the DMM, but appear spatially separate in the ventral region of the limb bud.

**Fig 4:**
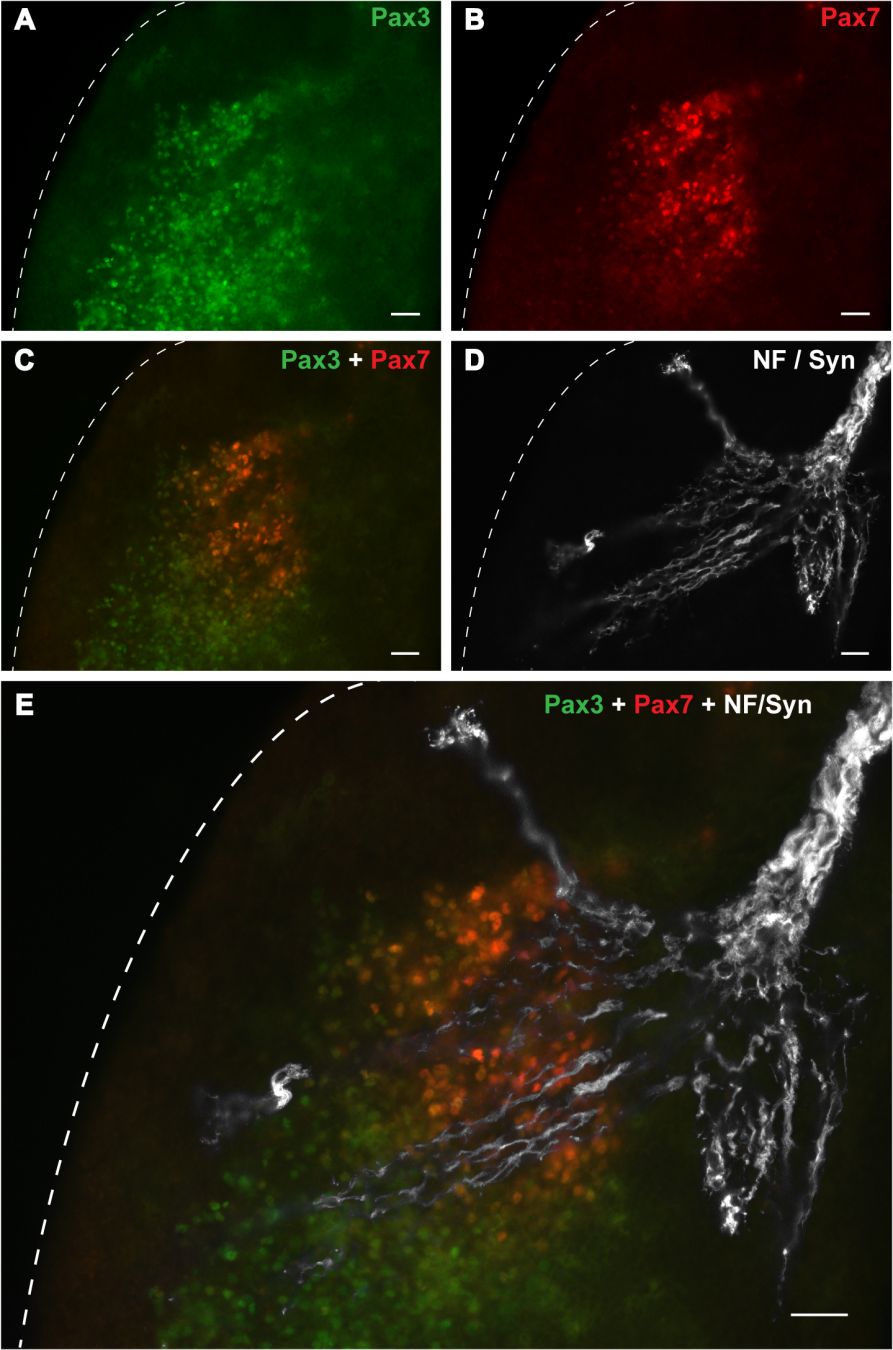
Spatial relationship between innervation and MPCs. 10 μm transverse sections of E14.5 rat hindlimb showing immunohistochemistry with monoclonal Pax3 (**A**) and polyclonal Pax7 (**B**) antibodies. A merge of the red and green channels (**C**) shows the Pax7-positive MPCs arising in the proximal/medial border of the Pax3-positive dorsal muscle mass (DMM). A greyscale image of nerve immunostained with neurofilament (NF) and synaptophysin (syn) (**D**) within an immediately adjacent section has been overlaid to show the relationship between the Pax3-positive MPCs, the Pax7-positive MPCs and the developing innervation (**E**). The dashed line indicates the dorsolateral margin of the limb bud. Scale bars = 50 μm.

The relationship between nerve outgrowth and Pax3- and Pax7-positive MPCs was examined more closely using dual labelling with monoclonal Pax3 and polyclonal Pax7 antibodies at E14.0 (Fig 4). The nerve was immunostained separately in the immediately adjacent section of the series (grey—Fig 4D) and digitally overlaid to the Pax3/Pax7 image (Fig 4E). The compound image confirmed that Pax7-positive MPCs first appear in the dorsal muscle mass in the proximal zone closely allied to the region of innervation, while Pax3-positive MPCs extend much further distally.

### Dynamics of Pax3-positive and Pax7-positive MPCs during early myogenesis in the rat hindlimb

**Fig 5:**
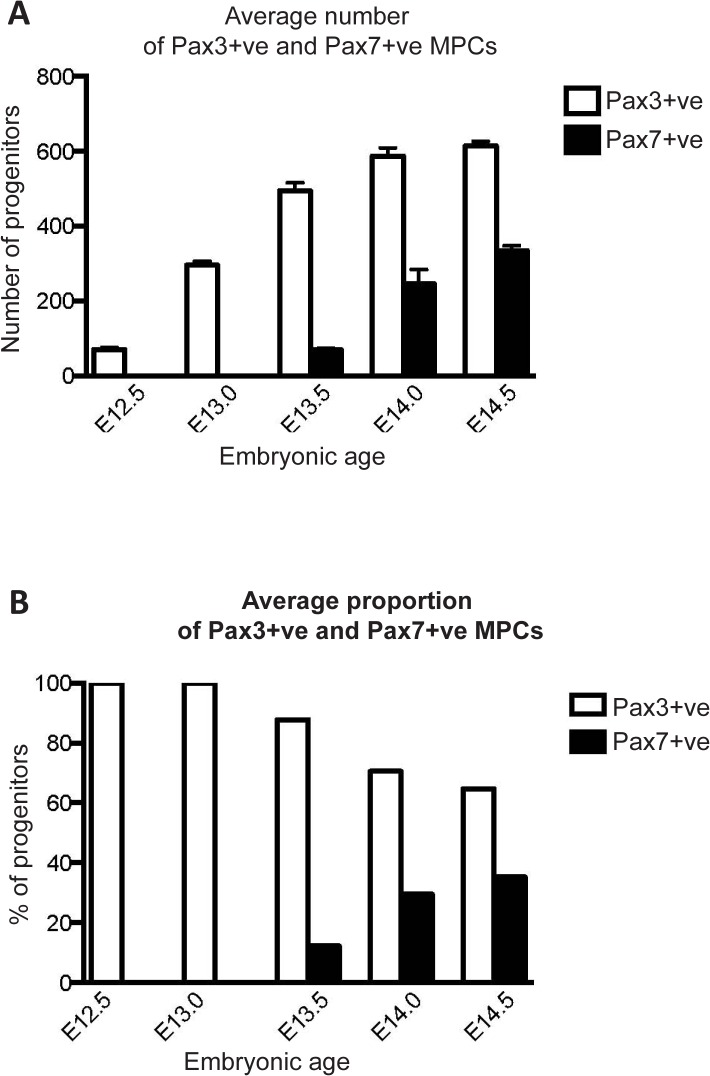
Quantitative analysis of MPC populations between E12.5 and E14.5. **A.** Mean (+/-SEM) numbers of Pax3- and Pax7-positive MPCs in rat hindlimbs sampled at half-day intervals from E12.5 to E14.5. All sections were stained by immunohistochemistry and analysed using systematic counts across all muscle masses in the entire limb section, n = 6 embryos at each age (see Materials and Methods). **B.** Proportions of Pax3- and Pax7-positive MPCs in rat hindlimbs sampled at half-day intervals from E12.5 to E14.5. The sampled mean (+/-SEM) numbers of Pax3 and Pax7-positive MPCs (**A**) are expressed as proportions relative to the total population sampled.

The quantitative dynamics of the Pax3-positive and Pax7-positive cell populations also differ over time. Initially, all myogenic precursors in the hindlimb at both E12.5 and E13.0 are immunoreactive for Pax3 only (100% of cells counted) (Fig 5A and 5B). By E13.5, the first Pax7-positive MPCs are seen, but they are few (approximately 50 cells across 5 sections, comprising about 10% of the total MPCs counted in a single limb bud). In contrast, the dorsal and ventral masses contain approximately 500 Pax3-positive cells, making up approximately 90% of the MPCs counted. Pax3-positive MPC numbers continue to rise, reaching approximately 600 cells counted and 65% of the total cells counted by E14.5 (see Fig 5A and 5B). From E13.5 to E14.5, Pax7-positive MPCs also increase steadily in number, but at a faster rate than Pax3-positive MPCs, so that they comprise a progressively larger proportion of the MPCs present in the limb (Fig 5B), reaching 30% of total MPCs by E14.5. At later times, the Pax7-positive MPC numbers continue to increase, whilst the Pax3-positive population decreases in number until Pax3 expression is lost completely (E16.0, data not shown).

## Discussion

### Timing of nerve entry into the limb muscle masses correlates with the onset of myogenic differentiation

The study described here correlates the outgrowth of the nerve into the rat hindlimb muscle mass with myogenic differentiation (see Table 2). It shows that Pax3-positive MPCs enter the limb bud first, followed a day later by the nerve at the time and place of the onset of muscle differentiation. The current literature does not describe these developmental events in any great detail and the general assumption is that nerve only grows into the developing limb well after primary myogenesis has started [17, 28, 39–41]. One early study analysed specifically the growth of nerves into the hindlimb of the rat using the protein GAP-43 to mark the growing nerves [42]. They found that axons grow into the limb at E14 which is similar to our results (depending on their exact definition of E0.5, which is not specified) however, they did not stain for myogenic markers and concluded that the nerves are not in contact with muscles before E15. In contrast, we find that by E15, muscle cells had been in contact with the nerve for more than 24 h and already bore clusters of AChR, reflecting a functional innervation [27, 28, 43].

**Table 2:** Summary of nerve and myogenic cells state at different developmental stages

Rat Hindlimb	E12.5	E13.0	E13.5	E14.0	E14.5	E15.0
**Nerve: NF+Syn**	Motorneurons grow out of neural tube	Ventral ramus passes somite	Nerve enters muscle masses*	Nerve in muscle masses*	Nerve in muscle masses	
**Muscle mass: Pax3**	In proximal limb	In muscle masses	In muscle masses*, slightly reduced in numbers	In muscle masses*, reduced compared to E13.5	In muscle masses, numbers about 60% of E13.0	
**Muscle mass: Pax7**	Absent	Absent	Few cells in proximal limb near ingrowing nerve*	Present near ingrowing nerve*	Present near ingrowing nerve, numbers about 250% of E13.5	
**Muscle mass: MHC**	Absent	Absent	Few cells in proximal limb near ingrowing nerve*	Present near ingrowing nerve*	Present near ingrowing nerve	
**AChR pattern**				Diffuse on cells in muscle masses	Some foci observed	Many foci observed

All descriptions refer to rat hindlimb.

*Marks descriptions also seen in mouse hindlimb at equivalent stages.

AChR—Acetylcholine receptor, MHC—myosin heavy chain, NF—neurofilament, syn—synaptophysin

The time course of nerve outgrowth in the rat and mouse limb, as presented here, exhibits one profound difference from nerve outgrowth in the chick limb. During embryonic development in the chick limb, motor axons grow out slightly before the sensory axons [44, 45], just as observed in the rat embryo, but when these axons reach the plexus region at the base of the limb they wait for approximately 24 hours, until the limbs become sufficiently mature, before entering the limb [19, 20]. This temporal delay of nerve entry is not seen in the rat and mouse limb, as our analysis at 0.5 embryonic day intervals shows no hesitation in the outgrowth of neurites. This distinct difference in nerve and limb development between the chick and mammals suggests that the early mechanisms of muscle innervation may have essential differences.

Early studies looking at the role of nerve during muscle development in the limb showed that when chick, mouse and rat embryos were denervated in the early embryo, primary myotube formation was unaffected, but there was a reduction in the numbers of secondary myotubes produced at later times [15, 17, 46, 47]. The conclusion was that early myogenesis is independent of neural influence. Surprisingly, in this study, we found a close temporal correlation between initial nerve contact with the pool of myogenic cells and the onset of myogenic differentiation. In fact, within the presumptive muscle mass, the first myogenic cells expressing MHC were seen close to the invading neurites, and the timing of their formation was temporally correlated with initial invasion of the muscle mass by nerve growth cones. In most studies of the impact of denervation on myogenesis, the denervation was performed relatively late during myogenesis, after the onset of formation of primary myotubes (e.g. [15, 46]) and, according to our results, well after nerve entry. Other analyses of mutant aneural mouse embryos were done even later, during primary and secondary myogenesis (e.g. [48, 49]). Thus, the possibility of a very early interaction between nerve and the precursor cells of the early limb muscle masses has never been excluded. Our results demonstrate that such an interaction is plausible.

### Appearance of Pax7-positive MPCs correlates in time and space with the arrival of innervation

During the earliest invasion of MPCs into the limb, they express only Pax3; a day later, Pax7-positive cells are also seen in the proximal region of the central limb bud and they progressively begin to outnumber the dwindling number of Pax3-expressing cells. Later in development, Pax3-positive cells become rare in the muscle masses, while Pax7-positive cells rapidly increase in number (Table 2). These changes in the number of Pax3-positive and Pax7-positive cells reflect their different progression through the myogenic cascade. Some Pax3-positive cells differentiate into primary myotubes and turn on MRFs and MHC while others go on to express Pax7, thus increasing the number of Pax7-positive cells available to contribute to secondary myogenesis and to the self-renewing population that will later be the stem cells of adult muscles [50–52].

It is known that all Pax7-expressing cells previously express Pax3 [51] and that Pax7 is unable to replace Pax3 during the migratory phase of muscle development [53]. Thus our results are compatible with the possibility that there are two different populations of muscle precursor cells migrating into the limb (as previously discussed in [8]): (i) a population of Pax3-positive/Pax7-negative cells that populates the early limb bud and that is exhausted during primary myogenesis, and (ii) a Pax3-positive population that migrates into the proximal limb bud in close proximity to the nerve, turns on Pax7, and becomes the precursor population of secondary myoblasts and satellite cells. An alternative interpretation of our data is that there is only one population of muscle precursor cells but that at the time of arrival of the nerve within the limb bud at E13.5, Pax7 expression is turned on in a subset of MPCs in the limb and these give rise to secondary myoblasts and satellite cells. In both scenarios, the nerve is, spatially and temporally, in a position to initiate and/or influence the pool of Pax7-positive cells which is consistent with the effect of denervation on secondary myogenesis [15, 47].

### Potential early signaling events between nerve and MPCs

Any potential role of nerve in influencing myogenesis would have to be mediated by a signal between nerve and myogenic cells. As the prime pathway for neuromuscular communication is via acetylcholine and its receptor, this pathway needs to be considered first. Early work using nerve-muscle co-cultures and electrophysiology [38] showed that the motor nerve growth cone continuously releases ACh as it grows; and in the chick embryo differentiating myoblasts produce acetylcholinesterase [54]. Further, Entwistle et al., 1988 [55] have shown that blockade of AChR markedly delays formation of myotubes in cultures of chick myoblasts. Early myoblasts have been shown to have functional receptors for acetylcholine; therefore the propensity for nerve-muscle interactions is present very early in myogenic development in culture [39, 56]. In the study presented here, AChR could only be diffusely detected on the limb myogenic cells 12h after nerve entry and after the appearance of the first myocytes. It is thus not likely that Ach is used to trigger primary myogenesis in mammalian embryos, which is consistent with the literature. However, it is possible that the ACh released by the nerve could be a signal for secondary myogenesis. However, the sensitivity of detection of AChR is low with immunohistochemistry, so our observations do not exclude a possible role for this pathway in regulating early myogenesis.

Another likely signal is one of the neuregulins. Previous studies have shown that by blocking glial growth factor 2, a member of the neuregulin family secreted by motor axons, or its receptor ErbB3 in rat myoblast cultures, expression of MHC and myotube formation is inhibited [57, 58]. Recent observations also show that neural crest cells migrating alongside the myotome release Neuregulin1, which, via the receptor ErbB3, maintains proliferation in the migrating myoblasts and inhibits differentiation [12]. Neuregulin1 is therefore very important for the early stages of myogenesis in the myotome, prior to the presence of AChR clusters [32], raising the possibility it might have a similar role in the limbs. In the mutant Wnt1-Cre:Pax3^ERD/+^ where most NCCs fail to migrate [12], myogenic differentiation in the limbs is severely impaired (myogenin and MyoD are almost absent) but it is unclear whether this lack of differentiation is due to a loss of MPCs in the limb or to a direct effect on their differentiation. In either case, the lack of NCCs has a great impact on myogenesis in the limb. Interestingly, when Schwann cells and sensory neurons–both derived from the NCCs and expressing neuregulin1 [59]–do not develop in mutant mouse embryo HB9^-/-^, the development of the diaphragm seems normal [49]. However, a specific analysis of early myogenic differentiation was not done.

Muscle tissue is part of a larger system which, to be set up properly, needs tight coordination between several tissues during development. It is becoming increasingly clear that myogenic cells and connective tissues differentiate in synchrony and that this synchrony involves communication events between the two [11]. Muscle and nerves also need to develop in a synchronized way as has been documented for late stages of myogenesis [28, 60]. The major contribution of the present study, showing the presence of nerve in the limb muscle mass at the time of onset of primary myogenesis, indicates that crosstalk between nerve and myogenic cells in mammalian embryos may occur much earlier than previously appreciated. Thus the interesting possibility arises that a three-way crosstalk between connective tissues, myogenic cells and nerves may play a role in setting up the musculo-skeletal system in mammals.

## Supporting Information

S1 Fig Nerve outgrowth in rat forelimb in relation with differentiated cells and MPCs. 10 μm transverse sections of rat forelimb at stages E12.5 (**A**, **B**) and E13.0 (**C**, **D**) stained by immunofluorescence against neurofilament (NF) and synaptophysin (Syn) (green). At stage E12.5, nerve staining is combined with antibodies against MHC and Pax3 to mark differentiated muscle cells and MPCs respectively (**A**, **B**). **B** shows a higher magnification of the area framed in **A**. At E13.0, two adjacent sections are stained to see the nerve (green as described above) and either Pax3 (red, **C**) or Pax7 (red, **D**). The events described in hindlimb occur half a day earlier in forelimb. The nerve invades the muscle at the very beginning of differentiation and Pax7 appears later than Pax3, correlated with the time of nerve entrance. DMM = dorsal muscle mass, VMM = ventral muscle mass. Scale bar = 200 μm
(TIF)

S2 Fig Time course of nerves entering the limb in the mouse embryos in relation with MPCs and differentiated muscle cells. 10 μm transverse sections of mouse embryo at forelimb level, stage E11.0 (**A**-**E**) and E11.5 (**F**- **H**) all stained by fluorescent immunohistochemistry to mark the nerves (green) with antibodies against neurofilament (NF) and synaptophysin (Syn). **A** is also immunostained against Pax3 to mark the MPCs (red) and **B** is a high magnification view of the framed area in **A**. **C**-**E** and **F**-**H** are three adjacent sections marked for either Pax3, Pax7 or MHC (red) combined with nerve staining. As in the rat embryo, nerves enter the limb well after Pax3-positive MPCs have established the DMM and VMM (**A** and **B**). Pax7-positive MPCs appear later, and their appearance is temporally and spatially correlated with nerve entry into the limb bud (arrows in **D**, **G**). MHC-positive differentiated myocytes are also observed near the nerve (arrowheads in **E**, **H**). These results confirm the observations made in the rat embryo. Note: At E11.5, some non-specific staining of red blood cells occurs; these appear as bright red, circular structures in all sections. DMM = dorsal muscle mass, VMM = ventral muscle mass, NT = neural tube, My = myotome. Scale bars = 100 μm (**B**-**H**); 200 μm (**A**).
(TIF)
