# Supplementary figures and images for: First Neuromuscular Contact Correlates with Onset of Primary Myogenesis in Rat and Mouse Limb Muscles
Source: PLoS One. 2015 Jul 24;10(7):e0133811. doi: 10.1371/journal.pone.0133811 (PMC4514893; doi:10.1371/journal.pone.0133811)

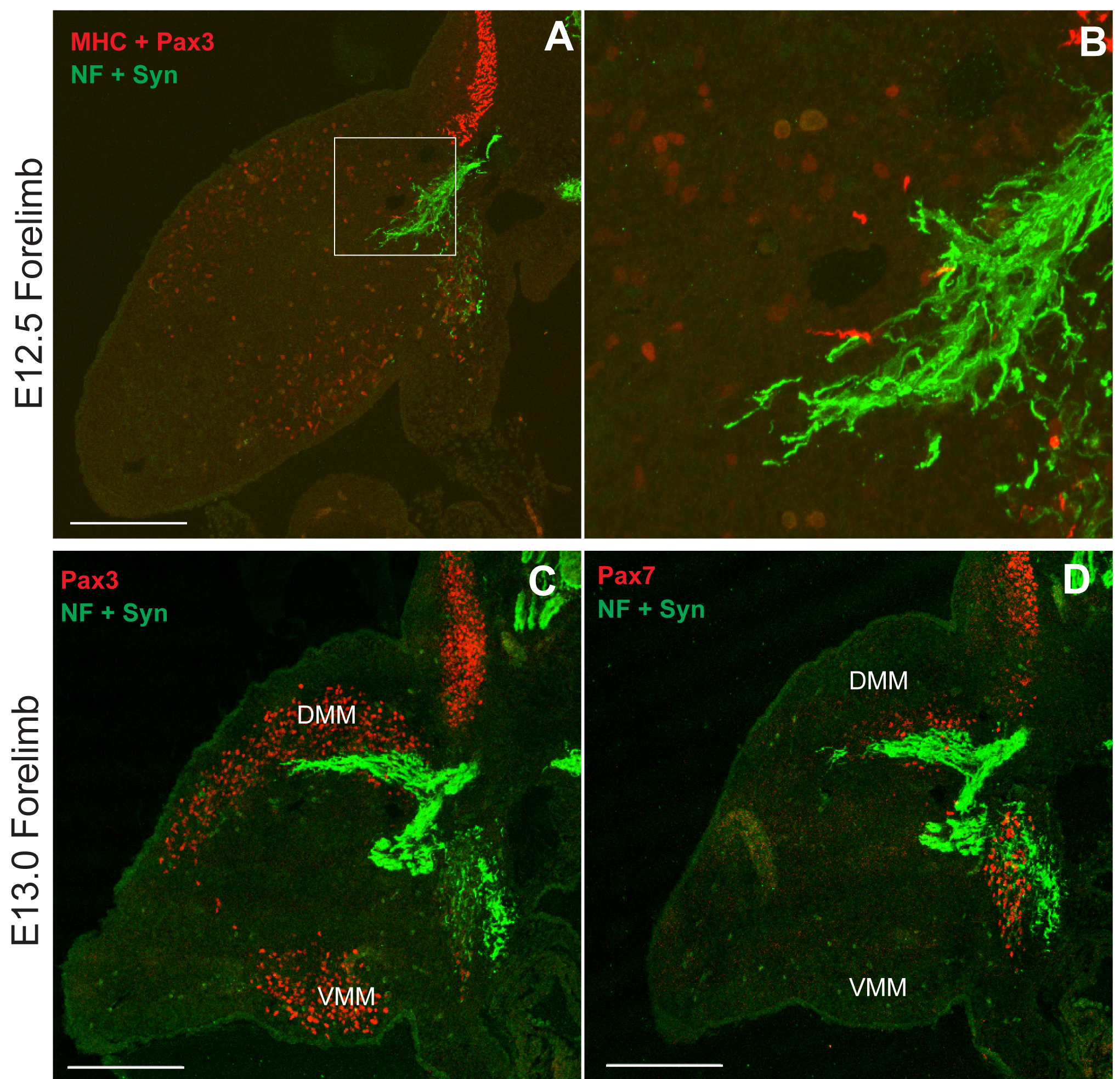

Supplement: S1 Fig — 10 μm transverse sections of rat forelimb at stages E12.5 (A, B) and E13.0 (C, D) stained by immunofluorescence against neurofilament (NF) and synaptophysin (Syn) (green). At stage E12.5, nerve staining is combined with antibodies against MHC and Pax3 to mark differentiated muscle cells and MPCs respectively (A, B). B shows a higher magnification of the area framed in A. At E13.0, two adjacent sections are stained to see the nerve (green as described above) and either Pax3 (red, C) or Pax7 (red, D). The events described in hindlimb occur half a day earlier in forelimb. The nerve invades the muscle at the very beginning of differentiation and Pax7 appears later than Pax3, correlated with the time of nerve entrance. DMM = dorsal muscle mass, VMM = ventral muscle mass. Scale bar = 200 μm (TIF) [file pone.0133811.s001.tif]

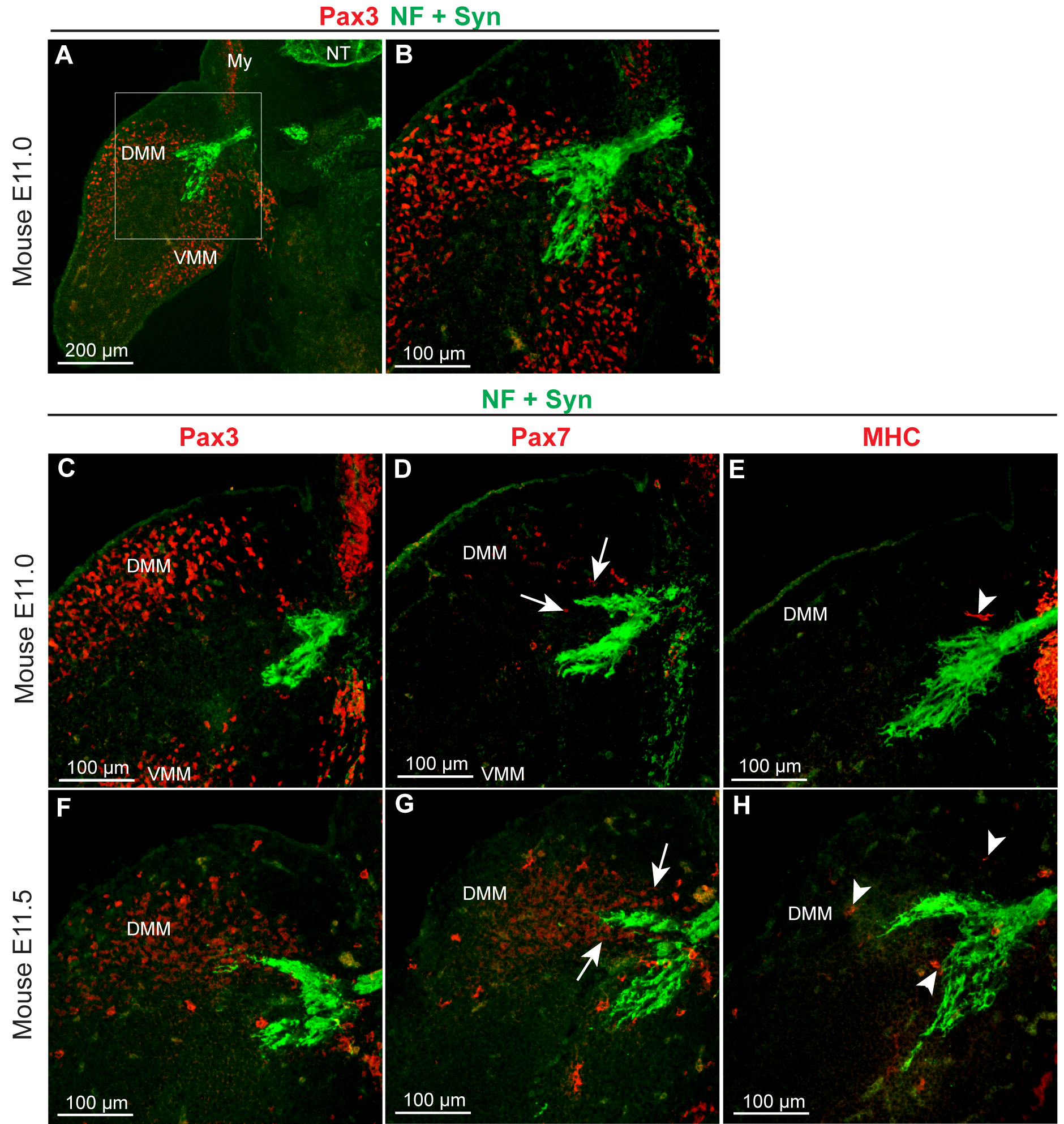

Supplement: S2 Fig — 10 μm transverse sections of mouse embryo at forelimb level, stage E11.0 (A-E) and E11.5 (F- H) all stained by fluorescent immunohistochemistry to mark the nerves (green) with antibodies against neurofilament (NF) and synaptophysin (Syn). A is also immunostained against Pax3 to mark the MPCs (red) and B is a high magnification view of the framed area in A. C-E and F-H are three adjacent sections marked for either Pax3, Pax7 or MHC (red) combined with nerve staining. As in the rat embryo, nerves enter the limb well after Pax3-positive MPCs have established the DMM and VMM (A and B). Pax7-positive MPCs appear later, and their appearance is temporally and spatially correlated with nerve entry into the limb bud (arrows in D, G). MHC-positive differentiated myocytes are also observed near the nerve (arrowheads in E, H). These results confirm the observations made in the rat embryo. Note: At E11.5, some non-specific staining of red blood cells occurs; these appear as bright red, circular structures in all sections. DMM = dorsal muscle mass, VMM = ventral muscle mass, NT = neural tube, My = myotome. Scale bars = 100 μm (B-H); 200 μm (A). (TIF) [file pone.0133811.s002.tif]
